# Assessing the Impact of Colchicine on Pulmonary Fibrosis Using Imaging in Patients With Moderate‐to‐Severe COVID‐19 Pneumonia

**DOI:** 10.1155/carj/9923052

**Published:** 2026-01-07

**Authors:** Gulhan Ayhan Albayrak, Mustafa Ilteris Bardakci, Ugur Temel

**Affiliations:** ^1^ Pulmonology Department, Şişli Hamidiye Etfal Training and Research Hospital, University of Health Sciences Turkey, İstanbul, Turkey, akdeniz.edu.tr; ^2^ Department of Thoracic Surgery, Şişli Hamidiye Etfal Training and Research Hospital, University of Health Sciences Turkey, İstanbul, Turkey, akdeniz.edu.tr

**Keywords:** colchicine treatment, COVID-19 and pulmonary fibrosis, COVID-19 pneumonia, recovery from COVID-19 pneumonia

## Abstract

**Objective:**

Pulmonary fibrosis may develop in patients with severe clinical conditions, especially those with high inflammatory indicators. We aimed to investigate the 15‐month follow‐up clinical outcomes in patients with moderate‐to‐severe COVID‐19 pneumonia treated with colchicine and to evaluate its potential in preventing post‐COVID pulmonary fibrosis.

**Method:**

This study, beginning with a retrospective analysis of 1489 selected patients, and a follow‐up assessment of 155 patients conducted 15 months after hospital discharge. This study included 90 patients presenting with COVID‐19 pneumonia with severity ranging from moderate to severe who were treated with colchicine alongside standard care, and 65 patients who did not receive colchicine were included in the control group to serve as a comparator. Patients who received colchicine treatment were included in Group 1, and those that did not in Group 2.

**Results:**

From admission to the 15‐month follow‐up, patients receiving colchicine exhibited a significant regression in chest CT abnormalities when compared with the control group (*p* < 0.05). Additionally, 65.6% (*n* = 59) of the colchicine group experienced symptomatic improvement, significantly higher than 35.4% (*n* = 23) in the control group (*p* < 0.05). Ninety patients who received colchicine treatment had Stage 3, 4, or 5, and only 28 had Stage 3, 4, or 5 pulmonary fıbrosis at the end of 15 months. After 15 months of follow‐up, all 20 patients initially classified as Stage 5 who received colchicine remained at Stage 5 pulmonary fibrosis, whereas in the control group, 2 patients (3.1%) were fibrosis‐free, and the others presented with Stage 1 (*n* = 23; 35.4%), Stage 3 (*n* = 23; 35.4%), Stage 4 (*n* = 9; 13.8%), or Stage 5 (*n* = 8; 12.3%) pulmonary fibrosis.

**Conclusion:**

This study demonstrates that colchicine therapy might reduce pulmonary fibrosis progression in individuals affected by COVID‐19 pneumonia. Clinicians should consider colchicine treatment to prevent pulmonary fibrosis.

## 1. Introduction

COVID‐19 pneumonia was initially reported in Wuhan, China, in December 2019, and officially recognized on January 7, 2020. This highly contagious disease, caused by the novel coronavirus SARS‐CoV‐2, had not been previously identified in humans. Due to its rapid global spread and severe impact, the World Health Organization (WHO) declared it a pandemic on March 11, 2020, with the infection leading to substantial morbidity and mortality worldwide [1]. As of May 28, 2023, COVID‐19 pandemic has affected more than 767 million people and led to the death of more than 6.9 million people [2]. Based on the available evidence, risk factors for COVID‐19 pneumonia in adults range from demographic factors such as advanced age, male gender, and ethnicity to the presence of underlying diseases such as hypertension, cardiovascular disease (CVD), and chronic obstructive pulmonary disease (COPD) [3].

Neutrophils and macrophages are the primary cells involved in the pathogenesis of respiratory viral infections. An elevated neutrophil count has been identified as a factor associated with severe outcomes in individuals diagnosed with COVID‐19, and extracellular traps of neutrophils are major pathogenic factors in COVID‐19 pneumonia [4]. Furthermore, neutrophil extracellular traps contribute to cytokine storming by promoting IL‐6β secretion in macrophages through inflammatory activation that involves IL‐1 production and promotes a hyper‐coagulation state [5].

Colchicine is a readily available drug that suppresses the excessive function of neutrophils, monocytes, and macrophages [6]. Colchicine can inhibit viroporin E–mediated activation of inflammation during SARS‐CoV‐2 infection, which leads to disruption of IL‐1β production resulting in the disappearance related to IL‐6 and tumor necrosis factor‐alpha (TNF‐α) secretion and reduced uptake of neutrophils and macrophages [6, 7]. Colchicine also reduces the production of reactive oxygen species and antimicrobial peptide α‐defensin [6, 8]. Colchicine inhibits neutrophil‐platelet aggregation and increases protein C levels by emphasizing its antithrombotic properties [6, 9]. Colchicine is also an antifibrotic and cardioprotective drug and has been shown to have antiviral effects in Zika infections [8, 10]. The safety profile of colchicine at a dose of 0.5–2 mg per day has been proven by decades of observational studies and clinical practice [11].

Previous literature elaborated that several patients discharged after suffering from COVID‐19 pneumonia have persistent abnormalities in their chest computed tomography (CT) scans. Fibrosis is more likely to develop in patients with severe clinical conditions, especially in patients with increased levels of inflammatory indicators. Interstitial thickening, irregular interface, rough reticular pattern, and parenchymal band that occur during the disease process are thought to be determinants of pulmonary fibrosis [12]. This study aimed to analyze and compare the 15‐month follow‐up clinical outcomes in individuals with moderate‐to‐severe COVID‐19 pneumonia receiving treatment with colchicine, while also assessing its potential role in preventing post‐COVID pulmonary fibrosis.

## 2. Method

This retrospective study included 155 patients aged 18 years or older who were monitored at our institution’s COVID‐19 inpatient clinic between March 26, 2020, and July 30, 2022. All study procedures adhered to the ethical guidelines of both national and institutional human research committees, in accordance with the 1975 Helsinki Declaration, as amended in 2008. The study protocol received approval from the Ethics Committee of our institution (protocol number 2258). Due to the retrospective design, informed consent was not required from the participants.

### 2.1. Study Population

This study enrolled patients who tested positive for SARS‐CoV‐2 via PCR and/or SARS‐CoV‐2 IgM/IgG test, those diagnosed with COVID‐19 pneumonia both clinically and radiologically, and individuals with no other identifiable disease apart from COVID‐19 pneumonia, according to the COVID‐19 Field Guide issued by the Ministry of Health of Turkey [13]. Severe COVID‐19 pneumonia was defined based on the Ministry’s criteria as follows: (i) patients exhibiting symptoms such as fever, myalgia or arthralgia, cough, sore throat, nasal congestion, tachypnea (≥ 30/min), or SpO_2_ < 90% on room air; (ii) poor prognostic laboratory markers at admission, including lymphocyte count below 800/μL, CRP levels exceeding 40 mg/L, ferritin above 500 ng/mL, or D‐dimer greater than 1000 ng/mL; and (iii) evidence of bilateral diffuse pneumonia on chest X‐ray or CT scan [13]. This study had a cross‐sectional design, and patients who had applied to our pulmonology clinic between March 26, 2020, and July 30, 2022, were enrolled in this research study. Male patients were more commonly affected by COVID‐19 pneumonia ranging from moderate to severe and exhibited higher rates of intensive care unit admission.

### 2.2. Study Groups

**Figure 1:**
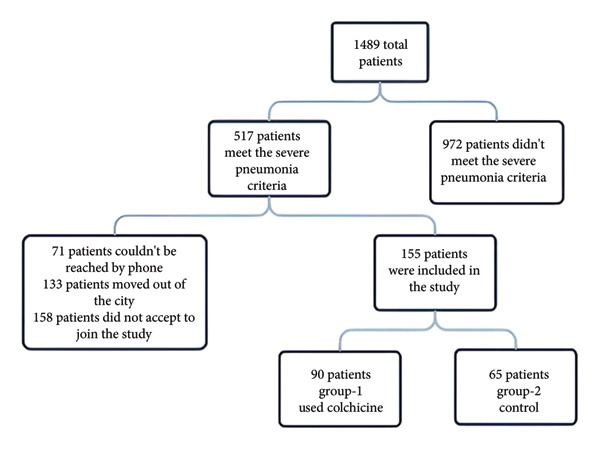
Patient participation scheme.

In the initial phase, the records of 1489 inpatients were retrospectively reviewed. Among them, 517 patients admitted with COVID‐19 pneumonia ranging from moderate to severe were contacted 15 months after discharge to participate in the study. However, 362 patients with incomplete data were excluded. The remaining 155 patients were enrolled in the prospective phase and invited for outpatient follow‐up 15 months post‐discharge. Of these, 90 patients who received colchicine were assigned to Group 1, while 65 patients receiving standard treatment comprised Group 2, serving as the control group. The patient flow diagram is presented in Figure 1.

### 2.3. Treatment Procedure

A total of 155 patients were assigned to two groups. Ninety patients in the colchicine group received standard care plus colchicine, while 65 patients received standard care alone. Standard care included COVID‐19 antiviral agents, corticosteroids, antithrombotic therapy, and oxygen supplementation. In line with the guidelines issued by the Turkish Ministry of Health at that time standard medications comprised favipiravir (200 mg, 2 × 8 tablets on day 1, followed by 2 × 3 tablets for 5 to 10 days), high‐dose intravenous steroids (prednisolone 30–100 mg daily), and low‐molecular‐weight heparin (enoxaparin sodium 1.5 mg/kg subcutaneously). Paracetamol (10–15 mg/kg orally) and antibiotics, including oral fluoroquinolones such as levofloxacin (500 mg/day) or moxifloxacin (400 mg/day), were administered as needed. Oxygen therapy was initiated when SpO_2_ fell below 91%, using conventional methods with nasal cannula or face mask at flow rates of 5–8 L/min, or with oxygen reservoir masks at 9–15 L/min. Colchicine was administered orally at 0.5 mg every 12 hours until discharge or for 10 days; for patients weighing less than 70 kg, the daily dose was 0.5 mg. Clinical records and laboratory results of all patients were reviewed [2, 13].

### 2.4. Inclusion and Exclusion Criteria

The study enrolled male and female inpatients aged 18 years or older who were diagnosed with moderate‐to‐severe COVID‐19 pneumonia and received 0.5 mg of oral colchicine in addition to standard COVID‐19 treatment protocols. Patients were excluded if they were under 18 years of age, had immunodeficiency, active malignancy, known colchicine hypersensitivity, severe heart failure, pulmonary fibrosis, or were pregnant or breastfeeding.

### 2.5. Patient Examination and Imaging

All participating patients underwent a thorough physical examination and routine laboratory tests. After obtaining informed consent, patients were evaluated using chest CT. The initial chest X‐ray findings recorded upon their initial hospital admission were compared with those of follow‐up chest CT scans obtained 15 months later. A total of 155 patients underwent nonenhanced chest CT for this study.

### 2.6. CT Protocol

Imaging was performed during a single inspiratory breath‐hold using a CTPA protocol at 100‐kV peak (kV[p]) and 20 effective milliampere‐seconds (eff mA·s). Axial images were acquired with a 2‐mm slice thickness using a 512 × 512 pixel matrix. For analysis, mediastinal window settings (width 400 HU; level 100 HU) and lung window settings (width 1500 HU; level −500 HU) were applied [1].

### 2.7. Radiological Assessment

All physicians participating in the study reached a consensus to classify CT findings into five categories, based on tomography features, interstitial changes, and estimated percentage of fibrotic areas. The images were independently evaluated by two radiologists who were blinded to the patients’ clinical information [1].

CT findings were categorized for the assessment of pulmonary fibrosis as follows:

(i) Stage 1: Normal, (ii) Stage 2: Predominantly involving ≤ 10% of surface area of medial segment of the lower lobe, (iii) Stage 3: Reticulation and traction bronchiectasis involving predominantly 10−50% of surface area of the medial segment of the lower lobe (iv), Stage 4: Reticulation and traction bronchiectasis, involving > 50% surface area of all lobes, and (v) Stage 5: Diffuse reticulation, traction bronchiectasis, and honeycombing involving > 50% surface area of all lobes [1].

### 2.8. Statistical Analysis

Data for this study were analyzed using IBM SPSS Statistics for Windows, version 23.0 (IBM Corp., Armonk, NY). Categorical variables were presented as frequencies and percentages, while continuous variables were reported as medians with minimum and maximum values. The Kolmogorov–Smirnov test was employed to assess the normality of the data. Comparisons between two groups were conducted using the independent samples *t*‐test for normally distributed variables and the Mann–Whitney *U* test for variables that did not meet normality assumptions. Categorical data were analyzed with either the chi‐square test or Fisher’s exact test. Logistic regression analysis was performed to identify risk factors associated with adverse tomography findings. A *p* value of less than 0.05 was considered statistically significant.

## 3. Results

This research involved an initial retrospective review of 1489 patients, followed by a prospective 15‐month follow‐up of the 155 eligible patients after hospital discharge. The study group (Group 1) included 90 patients with moderate‐to‐severe COVID‐19 who received colchicine in addition to standard care, while the control group (Group 2) comprised 65 patients who received standard care alone.

Group 1 consisted of 41(45.6%) female and 49 (54.4%) male, and Group 2 comprised 16 (24.6%) female and 49 (75.4%) male patients. The median (min–max) age of the 155 patients participating in the study was 58 (27–91) years. The median (min–max) ages of Group 1 and 2 patients were 60 (27–91), and 55 (30–82) years, respectively. The median (min–max) duration of hospital stay was 13 (7–69) days. Thirty‐five patients were treated as inpatients in intensive care units. Twenty‐two (24.4%) patients in Group 1 received intensive care treatment for an average of 13.5 days. Thirteen (20.0%) patients in Group 2 received intensive care treatment for an average of 11 days. The demographic characteristics of patients in the colchicine and control groups are presented in Table 1.

**Table 1:** The demographic characteristics of patients in the colchicine and control groups.

Variables	Total (*n* = 155)	Group 1 colchicine treatment group (*n* = 90)	Group 2 control group (*n* = 65)	*p*‐value
Age median (min‐max) (years)	58 (27–91)	60 (27–91)	55 (30–82)	0.0.087
Gender				0.0.008
Female, *n* (%)	57 (36.8)	41 (45.6)	16 (24.6)	
Male, *n* (%)	98 (63.2)	49 (54.4)	49 (75.4)	
Hospital stay, median (min‐max)	13 (7–69)	15 (7–69)	11 (7–42)	0.0.014
Intensive care unit, *n* (%)	35 (22.6)	22 (24.4)	13 (20)	0.0.647
Intensive care unit stay (days), median (min‐max)	12 (0–68)	13.5 (0–68)	11 (6–32)	0.0.259

The laboratory parameters of patients in the colchicine and control groups are presented in Table 2. D‐dimer levels were significantly elevated in the colchicine group (*p* = 0.006), whereas LDH (*p* = 0.42), WBC (*p* = 0.003), neutrophil (*p* = 0.01), and platelet counts (*p* < 0.001) were significantly elevated in the control group.

**Table 2:** Distribution of laboratory parameters of the patients in the colchicine and control groups.

Variables	Total (*N* = 155)	Group 1 colchicine treatment group (*n* = 90)	Group 2 control group (*n* = 65)	*p*‐value
	Median (min–max)	Median (min–max)	Median (min–max)	
Glucose (mg/dL)	122 (86–409)	121 (86–364)	124 (90–409)	0.181
Urea (mg/dL)	31 (13–141)	32 (13–95)	30 (14–141)	0.665
Creatinine (mg/dL)	0.9 (0.4–98)	0.8 (0.4–2.2)	0.9 (0.6–98)	0.142
Alanine Aminotransferase (U/L)	27 (7–149)	26.5 (9–149)	28 (7–110)	0.386
Aspartate Aminotransferase (U/L)	33 (11–272)	32 (11–272)	35 (13–177)	0.304
Lactate dehydrogenase (U/L)	311 (116–882)	325 (136–882)	301 (116–740)	0.042
D‐Dimer (μg/L)	530 (43–15101)	422 (200–15101)	593 (43–2460)	0.006
C‐reactive protein (mg/L)	66 (1–341)	72 (1–341)	65 (2–303)	0.405
Ferritin (μg/L)	319 (5–3024)	299 (5–3024)	349 (14–1983)	0.593
White blood cell (× 10^3^/μL)	6.4 (2.2–26.4)	7.1 (2.2–26.4)	6 (3.4–13.8)	0.003
Neutrophil (× 10^3^/μL)	4.9 (1.5–1071)	5.5 (1.5–1071)	4.3 (2.1–12.4)	0.010
Neutrophils (%)	75.9 (48–94.2)	76.9 (49.3–94.2)	74 (48–91)	0.259
Lymphocytes (× 10^3^/μL)	1.1 (0.2–8.1)	1.1 (0.2–8.1)	1 (0.4–3.6)	0.157
Lymphocytes (%)	16 (2.1–44)	15.8 (2.1–28)	18 (4–44)	0.602
Neutrophil‐to‐Lymphocyte ratio	4.7 (1.1–43)	5 (1.1–43)	4.1 (1.1–22.6)	0.419
Platelets (× 10^3^/μL)	186 (61–809)	200 (89–809)	169 (61–414)	< 0.001

### 3.1. Chest CT Findings at 15‐Month Follow‐Up

An analysis of the follow‐up chest CT scans revealed that 30 (19.4%) patients had normal findings, while 125 patients (80.6%) presented with noteworthy pathological changes. Among 125 patients with abnormal CT findings, 57 patients exhibited minimal changes (Stage 2). More advanced fibrosis was observed in the remaining patients, distributed across three stages:

Stage 3: Forty‐three patients demonstrated medial segment of the lower lobe dominant reticulation and traction bronchiectasis, involving 10−50% of the lung surface area.

Stage 4: Twelve patients showed widespread involvement across all lobes, characterized by reticulation and traction bronchiectasis, affecting over 50% of the lung surface area.

Stage 5: The most severe fibrotic changes were identified in 13 patients, who displayed diffuse reticulation, traction bronchiectasis, and honeycombing across all lobes, with involvement exceeding 50% of the lung surface area.

Among the 90 patients who received colchicine, all initially presented with Stage 3, 4, or 5 pulmonary fibrosis; however, only 28 still exhibited Stage 3–5 disease at the 15‐month follow‐up period. From the set of 20 patients who had Stage 5 fibrosis and were given colchicine therapy, five remained at Stage 5 after 15 months. In this group, pulmonary fibrosis had either resolved (*n* = 28; 31.1%) or persisted at Stage 1 (*n* = 34; 37.8%), Stage 3 (*n* = 20; 22.2%), Stage 4 (*n* = 3; 3.3%), or Stage 5 (*n* = 5; 5.6%) by the conclusion of the follow‐up period. Conversely, in the control cohort, fibrosis had either resolved (*n* = 2; 3.1%) or persisted at stage 1 (*n* = 23; 35.4%), Stage 3 (*n* = 23; 35.4%), or Stage 4 (*n* = 9; 13.8%) after 15 months. Table 3 provides a comparison of chest CT findings between the study groups.

**Table 3:** Chest CT findings of the patients in the colchicine and control groups.

Variables	Total (*N* = 155)	Group 1 colchicine treatment group (*n* = 90)	Group 2 control group (*n* = 65)	*p*‐value
	*n* (%)	*n* (%)	*n* (%)	
Chest CT findings of COVID‐19 patients				0.050
Stage 3	40 (25.8)	27 (30)	13 (20)	
Stage 4	69 (44.5)	43 (47.8)	26 (40)	
Stage 5	46 (29.7)	20 (22.2)	26 (40)	
Chest CT findings of the control group				< 0.001
Stage 1	30 (19.4)	28 (31.1)	2 (3.1)	
Stage 2	57 (36.8)	34 (37.8)	23 (35.4)	
Stage 3	43 (27.7)	20 (22.2)	23 (35.4)	
Stage 4	12 (7.7)	3 (3.3)	9 (13.8)	
Stage 5	13 (8.4)	5 (5.6)	8 (12.3)	
Chest CT recovery (Hospitalization/Control Chest CT)	82 (52.9)	59 (65.6)	23 (35.4)	< 0.001

*Note:* Stage 1: Normal, Stage 2: Predominantly reticulation involving ≤ 10% surface area of the middle‐lower lobe. Stage 3: Predominantly reticulation + traction involving 10%–50% surface area of the middle‐lower lobe. Stage 4: Reticulation + traction involving more than 50% surface area of all lobes, Stage 5: Diffuse reticulation + traction + honeycombing involving more than 50% surface area of all lobes.

### 3.2. Comparison of Parameters

There was a statistically significant regression between chest CT results at the time of hospitalization and the end of 15 months after discharge for COVID‐19 inpatients receiving colchicine treatment relative to the control group (*p* < 0.05). The analysis showed that pathological CT findings of 59 (65.6%) patients who received colchicine treatment had resolved. In comparison, the rate of resolution of abnormal CT findings was statistically significantly lower in 23 (35.4%) inpatients who did not receive colchicine therapy (*p* < 0.05).

## 4. Discussion

Pulmonary fibrosis is a sequela of adult respiratory distress syndrome (ARDS). Approximately 40% of patients with COVID‐19 disease develop ARDS, and 20% of these cases are severe. Current evidence suggests that developing pulmonary fibrosis due to COVID‐19 disease after SARS and MERS may complicate SARS‐CoV‐2 infection. Colchicine is an anti‐inflammatory drug for treating gout, pericarditis, and familial Mediterranean fever. The mechanism of action of colchicine entails the activation and migration of neutrophils into lungs and disrupts the inflammasome complex necessary in the production of IL‐18 and IL‐1β. Colchicine has a direct anti‐inflammatory effect [14]. In this study, we examined the long‐term impacts of colchicine on the development of post‐COVID pulmonary fibrosis in patients receiving inpatient treatment in COVID‐19 clinics of our hospital. We compared 90 patients who received colchicine treatment with 65 control patients who did not. Regarding the outcomes of this study, pulmonary fibrosis was less common in patients who received colchicine treatment for 15 months after discharge.

In COVID‐19, the immune system can trigger increased inflammation and secondary hemophagocytic lymphohistiocytosis or macrophage activation syndrome. This hypercytokinesia is characterized by ARDS, multiple organ failure, and sepsis. Activation of the NLRP3 (NOD‐like receptor pyrin domain‐containing protein 3) inflammasome represents considering a key contributor to this process. Chronic NLRP3 overactivity can lead to lung damage and fibrosis, cardiomyopathy, and consequences that may leave patients vulnerable to adverse effects of diabetes. A complex inflammation involving TNF‐α and interleukins develop during this process. Colchicine produces direct anti‐inflammatory effects through the inhibition of TNF‐α and IL‐6 production, preventing monocyte migration, and reducing matrix metalloproteinase‐1 secretion. Additionally, it suppresses cytokine and chemokine release and inhibits platelet aggregation in vitro. These potentially beneficial effects could reduce the COVID‐19 inflammatory storm associated with severe cases. Colchicine exhibits anti‐fibrotic properties by acting as a microtubule‐destabilizing agent. An in vitro investigation using human lung fibroblasts demonstrated that colchicine effectively modulates cellular inflammatory responses; it inhibited myofibroblast differentiation through the Rho/serum response factor (SRF) pathway. Additionally, Issak et al. have reported the application of colchicine for treating COVID‐19 pneumonia, highlighting its potential role in managing pulmonary inflammation, and evaluated its impact on inflammatory biomarkers and clinical outcomes [14].

Since colchicine has mild side effects and a potent anti‐inflammatory profile, studies were initiated with the hypothesis that colchicine could be an effective and a safe anti‐inflammatory option for the management of COVID‐19 disease [15]. With few exceptions, most investigations have targeted the treatment of COVID‐19 disease in the acute stage. There have been published studies on the effect of colchicine on long‐term fibrosis. Clinical studies have shown that low‐dose colchicine is effective not only in the treatment of acute gout but also in supporting long‐term management strategies [16]. Additionally, colchicine ameliorates the symptoms of inflammatory responses associated with COVID‐19 pneumonia. It reduces the frequency of onset of pulmonary infiltrates, headaches, and arthralgia. However, studies have shown that colchicine is used not as an initial or primary alternative to treat COVID‐19 disease but as an *“*off‐label*”* therapy in response to hyper‐inflammation caused by the release of cytokines [17]. Dorward et al. [18] reported that colchicine did not reduce recovery time in community‐based COVID‐19 patients at higher risk of complications. Similarly, based on available evidence, Mikolajewska et al. [19] concluded that colchicine is unlikely to provide significant benefits in terms of mortality or the clinical outcomes of hospitalized patients with moderate‐to‐severe COVID‐19 versus placebo or conventional treatment alone. Within the RECOVERY trial, treatment with colchicine was not linked to lower 28‐day mortality rates, length of hospital stay, progression to invasive mechanical ventilation, or overall risk of death among adults hospitalized with COVID‐19 pneumonia [20]. In one meta‐analysis, moderate‐quality evidence suggested no benefit from adding colchicine to the standard care protocol for patients with a confirmed COVID‐19 diagnosis [21]. In one study, colchicine treatment was associated with reduced CRP levels and the clinical severity among individuals with COVID‐19, whereas D‐dimer levels, all‐cause mortality, and rates of mechanical ventilation were unchanged. Therefore, further clinical studies are needed to adequately assess the effectiveness of colchicine in this group of patients [22]. Sultan et al. [23] reported that PaO_2_/FiO_2_ ratio improved more significantly in patients receiving budesonide compared to the supportive and colchicine treatment groups, and the average hospital stay was shorter when colchicine or budesonide was used as opposed to supportive care alone. They also reported a significant decrease in mortality rates with colchicine treatment compared to supportive care.

Immunomodulators (such as leflunomide and thalidomide) used for hematological and rheumatic conditions and colchicine, which prevents the NLRP3 inflammasome activation, have also been investigated for the treatment of COVID‐19 infection. In particular, colchicine is being tested in randomized controlled trials in Canada (COLCORONA study, NCT04322682), Italy (ColCOVID‐19 study, NCT04322565), and more recently, Greece (GRECCO‐19 study, NCT04326790) [24]. Low‐dose colchicine may be regarded as a safe and potentially effective option for managing and preventing cytokine storms in patients with SARS‐CoV‐2 infection, particularly when used alongside other therapeutic agents. Well‐designed clinical trials are warranted to further explore this potential [25].

Colchicine shortened the duration of supplemental oxygen therapy and hospitalization time. The treatment was associated with a favorable safety profile and high tolerability. With death an expected outcome, an outstanding reduction in the COVID‐19 mortality rate could not be achieved with colchicine treatment [26]. In a retrospective cohort study [27] in patients infected with COVID‐19, colchicine treatment was linked to a decrease in mortality and a faster recovery process. Hariyantu et al. [28] also recommended the routine use of colchicine for treatment modalities of COVID‐19 patients in their studies. According to Pordowlat et al. [29], colchicine can improve clinical outcomes and reduce pulmonary infiltration in patients with COVID‐19 disease, unless contraindications and precautions are taken into consideration. Colchicine treatment was associated with improved survival and accelerated recuperation in COVID‐19 patients, but it should be prescribed at the appropriate time and only for eligible cases.

In the current study, chest CTs were unremarkable in 19.4%, while they were abnormal in 80.6% of the cases. Chest CT changes were minimal (Stage 2) in 57 of the 125 patients with abnormal CT findings. Among the 90 patients who received colchicine treatment, Stages 3, 4, and 5 pulmonary involvement were initially observed, whereas only 28 patients exhibited Stage 3, 4, or 5 pathological CT findings at the 15‐month follow‐up. Notably, 5 of the 20 Stage 5 patients treated with colchicine still demonstrated Stage 5 pulmonary fibrosis at 15 months. Comparison of chest CT results between COVID‐19 inpatients who received colchicine and the control group revealed a marked improvement in pulmonary fibrosis in the colchicine‐treated group at the 15‐month follow‐up. Overall, abnormal CT findings resolved in 65.6% of patients receiving colchicine, whereas only 35.4% of patients in the control cohort demonstrated resolution of abnormal findings.

## 5. Limitations

The main limitation of this research stems from initial retrospective phase. Additionally, the authors could not reach all patients under colchicine treatment. The fact that the duration of hospital stays, D‐dimer levels, WBC, and neutrophil counts were higher in the patients receiving colchicine treatment and the severity of the disease detected on admission chest CT scans was greater in the control group suggested that colchicine treatment was more effective in patients with high D‐dimer levels, WBC, and neutrophil counts.

## 6. Conclusion

The present study demonstrates that colchicine treatment may prevent the development of pulmonary fibrosis in patients with COVID‐19. Furthermore, evidence from previous research indicates that colchicine can reduce disease severity and mortality in patients with COVID‐19 pneumonia, highlighting its potential as an adjunctive therapeutic option. This study has shown that addition of colchicine to the treatment regimens used for COVID‐19 patients should be considered to help ameliorate the severe outcome of the disease, reduce mortality rates, and prevent pulmonary fibrosis from developing. In the light of the increasing information, it is foreseen that colchicine was essential in developing COVID‐19 treatment regimens.

## Ethics Statement

This study fully complies with the ethical standards stipulated in the Declaration of Helsinki. Participants were fully informed of the study’s purpose, procedures, risks, and benefits before inclusion. All participants provided written informed consent, and the study protocol (No. 2258; approved on 07.03.2023) was approved by the Ethics Committee of the University of Health Sciences Şişli Hamidiye Etfal Training and Research Hospital, Clinic of Chest Diseases, Istanbul, Turkey.

## Conflicts of Interest

The authors declare no conflicts of interest.

## Funding

The authors received no financial support for the research, authorship, and/or publication of this article.

## Data Availability

Data will be made available upon request to the corresponding author.
